# Humic field biostimulation as a sustainable agricultural practice to increase yield of main grains: evidence from on-farm trials

**DOI:** 10.3389/fpls.2025.1709876

**Published:** 2025-12-09

**Authors:** Juan Izquierdo, Osvin Arriagada, Gustavo García-Pintos, Rodomiro Ortiz, Martín García-Pintos, Marcelo García-Pintos

**Affiliations:** 1 Servicios para el Desarrollo Rural Integral y la Agricultura (SEDRA), Punta del Este, Uruguay; 2 Centro de Estudios en Alimentos Procesados (CEAP), Talca, Chile; 3 BIOCIS, Paysandú, Uruguay; 4 Department of Plant Breeding, Swedish University of Agricultural Sciences, Alnarp, Sweden; 5 BIOCIS, Montevideo, Uruguay

**Keywords:** barley, economic profitability, humic biostimulant, maize, rice, soybean, sustainable agriculture, wheat

## Abstract

**Introduction:**

Conventional agriculture relies heavily on synthetic fertilizers and pesticides, raising questions regarding its long-term sustainability. The use of biostimulants is an environmentally friendly approach to improving crop yields. However, most of these results have been obtained under controlled conditions, making it necessary to evaluate them under commercial production conditions. Therefore, this study aimed to assess the effect of a single foliar spray of a humic biostimulant (HB) on the yield and profitability of five main crops.

**Methods:**

448 On-farm strip trials (OFT) were conducted on commercial farms from 2014 to 2024 in the main production zones of Uruguay. The HB was applied at a critical stage of the growth cycle in soybean (R3–R4), rice (V5–V9), maize (V6), wheat (Z1.6), and barley (Z1.6). Yield responses and net economic returns were measured for each crop.

**Results and discussion:**

The HB increased the average yield of all five crops. The overall mean yield response was significant, ranging from 7.6% to 15.7% for rice and maize, respectively. In barley, HB showed greater effectiveness at lower-yielding sites, reaching ~4 t ha^−1^. In rice, a tendency towards a greater impact on low- and high-yield OFTs was observed. In contrast, HB had a relatively constant effect on maize, wheat, and soybeans. Based on empirical data (normal scenario), the net economic returns varied from US $85.1 to $122.0 ha^−1^ for wheat and maize, respectively. The probability of exceeding the break-even cost ranged from 83.4% to 89.1% for soybean and wheat, respectively. To our knowledge, this is the first study to demonstrate that a single foliar application of HB at a critical stage of development increases crop yield and profitability for farmers under OFT conditions over several years and at multiple sites. Therefore, this practice can be applied by Uruguayan farmers to sustainably increase yields and economic profits.

## Graphical Abstract

**Figure:**
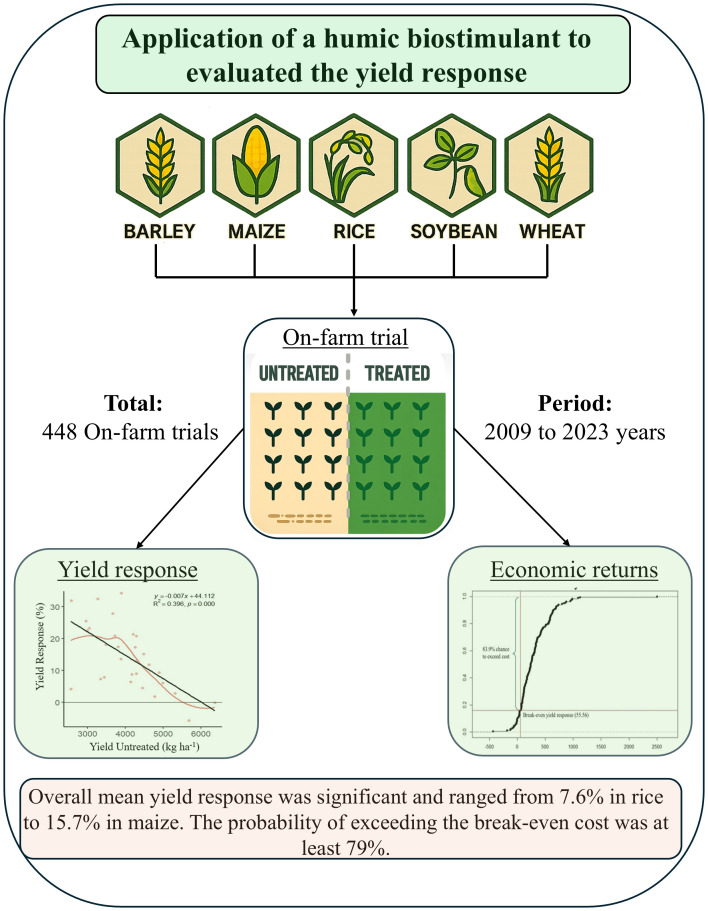

## 1 Introduction

It is estimated that by 2050, the world population will exceed 9.7 billion people; thus, the total global food demand is expected to increase by approximately 50%–60% (van Dijk et al., 2021). In this context, it is necessary to significantly increase agricultural production, where more agricultural land, fertilizer, water, and high-yielding crops will be necessary to ensure the food supply and reduce the yield gap (Burdon et al., 2020). Several factors contribute to yield gaps in important grain crops, including multicausal factors such as nutrient deficiencies and imbalances, water deficit or excess in the soil, suboptimal planting, soil problems, weed pressure, insect damage, diseases, lodging, and inferior seed quality (Beza et al., 2017). Maize, wheat, rice, soybean, and barley are among the top grain crops grown worldwide for food, fiber, feed, and fuel (FAOSTAT, 2023; https://www.fao.org/statistics/es). Four of them (maize, rice, wheat, and soybean) produce 64% of the agricultural calories for human or livestock consumption (Tilman et al., 2011). In this sense, increasing their production without adverse impacts on the environment remains a priority, particularly in the Global South (Yin and Ding, 2021). In Uruguay, from 2015 to 2022, the average annual planted area of rainfed crops was 1.7 million ha, including crops such as soybeans (59.50%), wheat (14.65%), barley (10.17%), maize (6.79%), and irrigated rice (8.88%), which are the country’s prominent field grains. The estimated mean yields during this period were 5.5 t ha^−1^, 3.6 t ha^−1^, 3.8 t ha^−1^, 2.1 t ha^−1^, and 8.6 t ha^−1^ for soybean, wheat, barley, maize, and rice, respectively (DIEA/MGAP, 2023). Considering that average yields in tropical and subtropical rainfed systems are commonly 50% or less of the yield potential (Lobell et al., 2009), there is ample scope to reduce the yield gap of these crops in Uruguay.

In the case of soybeans, yield losses of up to 43% have been previously reported due to summer drought stress and water deficiency between advanced fruiting and grain filling stages (Kobraei et al., 2011). This causes a significant reduction of up to 50% in yield (Giménez, 2014). In Uruguay has been reported a maximum achievable yield for soybeans of around 5 t ha^−1^ to 6 t ha^−1^ (Rizzo, 2018) and an estimated crop yield gap of 1.2 t ha^−1^–1.8 t ha^−1^ (Rizzo et al., 2021). Uruguay is among the top eight high-quality rice exporters worldwide (Rebollo et al., 2023). Rice grain yields are increasing but have not yet reached 80% of the estimated yield potential for irrigated rice of 15.8 t ha^−1^, thus resulting in a yield gap of 4.3 t ha^−1^ and 4.9 t ha^−1^ for eastern and northern Uruguay, respectively (Carracelas et al., 2019). In wheat, the average country yield gap was estimated as 1.5 t ha^−1^ ± 0.8 t ha^−1^ for the period 2009–2012 from a database of wheat-producing members of the Uruguayan Federation of Regional Consortia of Agricultural Experimentation (FUCREA). Malting barley after wheat is the second most important winter crop, thus playing a relevant role in crop rotation, with approximately 90% of the barley grain produced in Uruguay being locally processed and then exported as barley malt. In terms of yield, losses of up to 30% caused by excess winter rain have been observed, which is associated with a lower number of spikes (−21%) and smaller spike size (−15%) (Lamarca et al., 2010). Maize is a summer crop that is highly sensitive to water deficiency at the flowering or grain-filling stages, which are critical periods because hydric stress causes a decrease in grain yield (Andrade et al., 1996; Cakir, 2004; Giménez, 2012). The average national maize yield between 2014 and 2021 was 6 t ha^−1^ (DIEA/MGAP, 2023). Maize production in Uruguay is constrained by water availability, which is subject to large variability related to the El Niño Southern Oscillation (ENSO), thereby resulting in low yields for nearly 40% of the years within a 22-year climatic data series (Giménez et al., 2016).

Agricultural field crop production requires innovative approaches to optimize crop nutrition and yield. A promising and environmentally friendly approach is to incorporate biostimulants from sustainable resources (Rathor et al., 2023) to improve crop production by increasing the yield under both optimal and suboptimal growing conditions (Sible et al., 2021). Biostimulants, such as humic substances (HS), can be administered as foliar, soil, and seed treatments, making them highly adaptable to different types of crops. The action of humic substances (HS) in soil and plants has been recognized as relevant to sustainable agriculture, as a way to address major problems of food security, environmental pollution, and the economic costs associated with synthetic inputs while avoiding loss of crop yield and quality (Nardi et al., 2021). HS acts as an active component in the regulation of growth processes, nutrient transport systems, and primary and secondary metabolism (Canellas and Olivares, 2014; Nardi et al., 2017). Studies have indicated the ability of HS to promote plant growth when used in foliar applications (De Hita et al., 2020). It presents a mechanism of action related to greater nutritional, metabolic, and physiological responses unique to the plant, such as a larger root system surface and elongation of the primary roots. HS-induced root architecture modifications not only improve soil exploration and nutrient uptake but also modulate hormonal signaling between the root and shoot (Schaller et al., 2015). In fact, HS have hormone-like activity, such as auxins and cytokinins, which are essential for plant development and yield (Souza et al., 2022). Auxins play important roles in promoting root development, whereas cytokinins antagonize auxin responses to regulate the root development (Jan et al., 2024). A more developed root system increases auxin transport from the shoot to the root, which in turn stimulates cytokinin biosynthesis in the root tips (Kurepa and Smalle, 2022). These root-synthesized cytokinins are subsequently translocated to the shoots, where they promote cell division, leaf expansion, and shoot branching, ultimately contributing to increased crop yield (Zhao et al., 2021). This, in turn, is linked to the expression of transporters involved in nutrient uptake, for example, increasing root acid exudation and promoting plant interactions with growth-promoting microorganisms (Puglisi et al., 2009; Zandonadi et al., 2013; Olivares et al., 2017). In this context, the use of a humic biostimulant (HB) supports the local circular economy when obtained from HS vermicomposted extracts.

The integration of biostimulants into agricultural practices largely depends on their economic importance compared to conventional practices, as their application may be economically beneficial for one farm and useless for another. To establish the economic efficiency of biologically active stimulants, economic-mathematical models based on linear optimization could be applied to assess not only the increase in profitability and profit of certain crops but also whether the profit of the farm as a whole is increasing (Sarov and Boevsky, 2021). Therefore, the benefits must be clearly demonstrated in the form of research results and educational and transfer programs that will focus on real data obtained from field experiments or on-farm trials. The economic effect of biostimulant application must be computed based on the value of the yield increase resulting from the use of biostimulants and the costs of their application (Kocira et al., 2020). The Uruguayan grain crop sector has incorporated innovations, inputs, and technological processes; however, long-term field research, including results validation with farmers, is necessary to extend the use of HB for managing field crop production sustainability. The use of HB is not yet part of the agronomic management of grain crops, and field foliar application could be implemented as an agricultural practice only after long-term validation at the farm level, where crop genetics and physiology, soil properties, and other environmental factors affect the interpretation of HB efficacy. In this sense, the yield response has been a useful approach for measuring the effect of new agronomic practices without considering crops and environmental factors (Laurent et al., 2019; Izquierdo et al., 2024). Hence, the objective of this study was to evaluate the yield response and economic return of a single foliar application of a humic biostimulant in on-farm trials (OFTs) of soybean, rice, maize, wheat, and barley grown in the main producing areas of Uruguay.

## 2 Materials and methods

### 2.1 On-farm trials

This research pursued OFT in farmers’ fields as a testing methodology for new products and practices (Laurent et al., 2019). The data from these tests can also be used to determine the economic profitability of new technologies (Laurent et al., 2020a; Lacoste et al., 2022). The OFT had two main levels of replication: the replicated strips (or random samples taken from treated and untreated field crop strips) within a trial, capturing within-trial variability, and the trial sites representing different environments and characterizing between-trial variability across regions (Laurent et al., 2019). A total of 448 OFTs on soybean (181), rice (103), maize (60), wheat (74), and barley (30) grain crops were installed in commercial farmers’ fields from 2009 to 2023, along the main production areas in 53 localities in 16 departments in Uruguay (**Supplementary Figure 1**). The management practices were decided on each OFT by each farmer, including cultivar, planting date, irrigation, seed and fertilizer rates, pest management, weed control, and harvesting practices. Therefore, the application of HB to the treated crop strip at each OFT was the only variation in management between the two treatments. Each OFT was considered an independent and unique replicate of the experiment. The number of OFTs for all crops (448) during the evaluation period ranged from two to 29 independent OFTs (see the Data Availability Statement section). The timing for the single HB application was R3–R4 in soybeans and V5–V9 in rice, as defined by previous results (Izquierdo and García-Pintos, 2021; Izquierdo et al., 2021, 2023, 2024). In the case of maize, it was V6, and for wheat and barley, it was Z1.6). The number of OFT per crop, period covered, spraying system, and phenological stage at the time of application are shown in **Table 1**. The spraying equipment belonged to farmers or companies that provided spraying services. In all cases, the ground and aerial spraying equipment was previously calibrated according to the manufacturer’s instructions to apply a dose of 4 L ha^−1^ and a water volume of 65 L ha^−1^ for ground application and the same dose and 15 L ha^−1^ for aerial application. In soybeans, maize, wheat, and barley, the terrestrial self-propelled sprayers had a working width of 18 m, and for rice, the application was made using aircraft with a working width of 65 m. All applications were performed in the early hours of the morning on windless days. In all OFTs, up to three HB-treated strips were randomly drawn from a paper grid and marked in the field before the spraying. Likewise, 15 5-m row segments were randomly selected for the HB-treated and untreated crop rows. The locations of these segments were marked in the field using colored flags in both untreated and treated fields (**Supplementary Figure 2**). Randomly selected plants from these segments were harvested at maturity to measure grain yield. Grain yield (kg m^−2^) adjusted to 14% humidity was recorded.

**Table 1:** Crop, number of trials, period covered, spraying system, and phenological state at application time of on-farm trials conducted in Uruguay.

Crop	Number of OFTs	Period covered	Spraying system	Phenological state at application time	Reference
Soybean	181	2014–2023	Terrestrial	R3–R4	Fehr et al. (1971)
Rice	103	2015–2023	Airplane	V5–V9	Velázquez et al. (2015)
Maize	60	2014–2019	Terrestrial	V6–V8	Li T. et al. (2022)
Wheat	74	2009–2023	Terrestrial	Z1.6	Zadok et al. (1974)
Barley	30	20017–2023	Terrestrial	Z1.6	Zadok et al. (1974)

### 2.2 The humic biostimulant

HB was obtained locally from wheat and maize crop residues mixed with horse and cow manure and vermicomposted for 6 months using the earthworm *Einsenia foetida* (Hamilton, 2017). The extraction, pH stabilization, and dilution of HB were carried out following agro-industrial methods under Uruguay’s license and production registry. The final product, PromoBacter^®^ (BIOCIS, Mercedes, Uruguay) had the following composition: total humic extracts 5.72% P/V; humic acids 4.05% P/V; fulvic acids 1.22% P/V; boron 0.1% W/V; auxins (IAA) 0.1 mg L^−1^–0.05 mg L^−1^; gibberellins (GA3) 0.5 mg L^−1–^2 mg L^−1^; cytokinin 0.01 mg L^−1^–0.05 mg L^−1^; amino acids 7 mg L^−1^–9.5 mg L^−1^; Enzymatic Activity: oxidase and transpeptidase. Density: 0.003 g mL^−1^; and pH: 6.8. The content of plant hormones such as auxins, gibberellins, and cytokinins is a result of the vermicomposting process without the addition of synthetic hormones. The humic biostimulant is registered with the Agricultural Services of the Ministry of Agriculture, Livestock, and Fisheries of Uruguay under number 270/022 B0-001. Furthermore, after successful yield tests at numerous sites in the state of Rio Grande do Sul, it was recently registered as an imported product from Uruguay by the Brazilian Ministry of Agriculture with the number RS006691-5.000001 on 9 October 2025.

### 2.3 Crop husbandry

Practices such as crop rotations, cover crops, minimum tillage, intercropping, and symbiotic associations are normally applied by Uruguayan farmers. The general management practices were decided at each site by the field owners and managers, including cultivar, planting date, irrigation, seed and fertilizer rates, pest management, weed control, and harvesting. Previous management, soil properties, weather conditions, and farmers’ individual choices, such as cultivars, vary across sites and years and may have resulted in different production and environmental outcomes. However, in general, farmers also follow the recommendations of the National Institute of Agricultural Research or producer associations, as in the case of rice and barley cultivation. The application timing and HB dose were established by the project coordinator for all OFTs for the five crops. Key characteristics of soils, cultivars used, and predominant agronomic management of on-farm trials are presented in **Supplementary Table 1**. In 2022, an intense drought during the summer affected the yields of maize and soybeans in Uruguay. The mean maximum temperature, evaporation, and rainfall data for February, March, and April on the south-west coast, the most important agricultural zone of Uruguay, during the 2014–2023 period, are presented in **Supplementary Table 2**.

### 2.4 Statistical analysis

The main objective was to quantify the effect of foliar-applied HB on grain yield compared to the common farmer practice of no HB application. For this, the mean effect sizes were calculated for each OFT to assess the treatment effects on grain yield using the yield ratio (a ratio of the mean yield obtained with the new management practice to the mean yield in the control), which is unitless and does not depend on yield units (Laurent et al., 2019, 2020a). The effect size for each paired treated and untreated trial was established using the natural logarithm of the response ratio (Equation 1) to normalize the data and facilitate statistical analysis as follows:

(1) $$Effect\;size=ln(\frac{Treated}{Control})$$

The overall mean effect sizes and their 95% confidence intervals (CI) were estimated using the mean effect size from all OFTs for each crop by bootstrapping 10,000 replicates in the ‘DescTools’ package (Signorell et al., 2021), with the CIs calculated using the normal approximation interval (type = “norm”). This approach offers an intuitive and graphic way to interpret treatment effects, since CIs provide a range of possible effect sizes in which the true treatment effect is likely to lie (Carey et al., 2022). In some cases, to aid interpretation, we back-transformed the effect sizes and associated CIs into percentages (%). The sample sizes (OFTs) for each crop were 181, 103, 60, 74, and 30 OFTs for soybean, rice, maize, wheat, and barley, respectively, in this study. A linear model was fitted to assess whether the mean yield response to biostimulant application varied significantly by crop and year. Because each OFT consisted of a single replicate, which prevented the estimation of within-site variability, its effect was excluded from the model. In addition, simple linear and Loess regressions were performed to assess the relationship between the yield and yield response of the treated and untreated crops. The regressions and figures were performed and drawn using RStudio software. Empirical cumulative distribution function were calculated and plotted using the ‘ecdf’ function in R. Empirical cumulative distribution function estimation was used to assess the break-even yield response and the probability of expected profit for the five crops as performed by Laurent et al. (2020b). The yield difference (yield obtained with the application of HB minus the yield of the control) was calculated for each OFT. The yield differences were ordered from lowest to highest to plot the cumulative probability using the ‘ecdf’ function in R. To calculate the break-even cost, we considered the cost of the product (US$ 14 ha^−1^), cost of applying the product, and selling price of the crop. We considered a product cost of US$ 14 ha^−1^, the terrestrial application cost of US$ 10 ha^−1^ in soybean, wheat, and barley, and an airplane application cost of US$ 17 ha^−1^ in maize and rice. The Uruguayan mean market prices of May during the period 2015–2024 were US$ 384.2 ton^−1^, 216.6 ton^−1^, 215.2 ton^−1^, 215.2 ton^−1^, and 199.8 ton^−1^ for soybean, rice, maize, wheat, and barley, respectively (DIEA/MGAP, 2024) (**Supplementary Table 3**). To assess how changes in market prices and/or application costs affect profitability, a sensitivity test with three scenarios was conducted. An unfavorable scenario involved a drop in the market price of the crop (mean price—1 SD) and an increase in the cost of the product and application (inflation of 4.5%). In the normal scenario, the mean price of the crop during the period and the actual costs of the product and application were considered. A favorable scenario considered an increase in both the price of the crop (mean price + 1SD) and the actual costs of the product and application. The break-even yield and chance of exceeding costs due to the application of HB on grain crops under market price variations were calculated.

## 3 Results

### 3.1 Yield response

**Figure 1:**
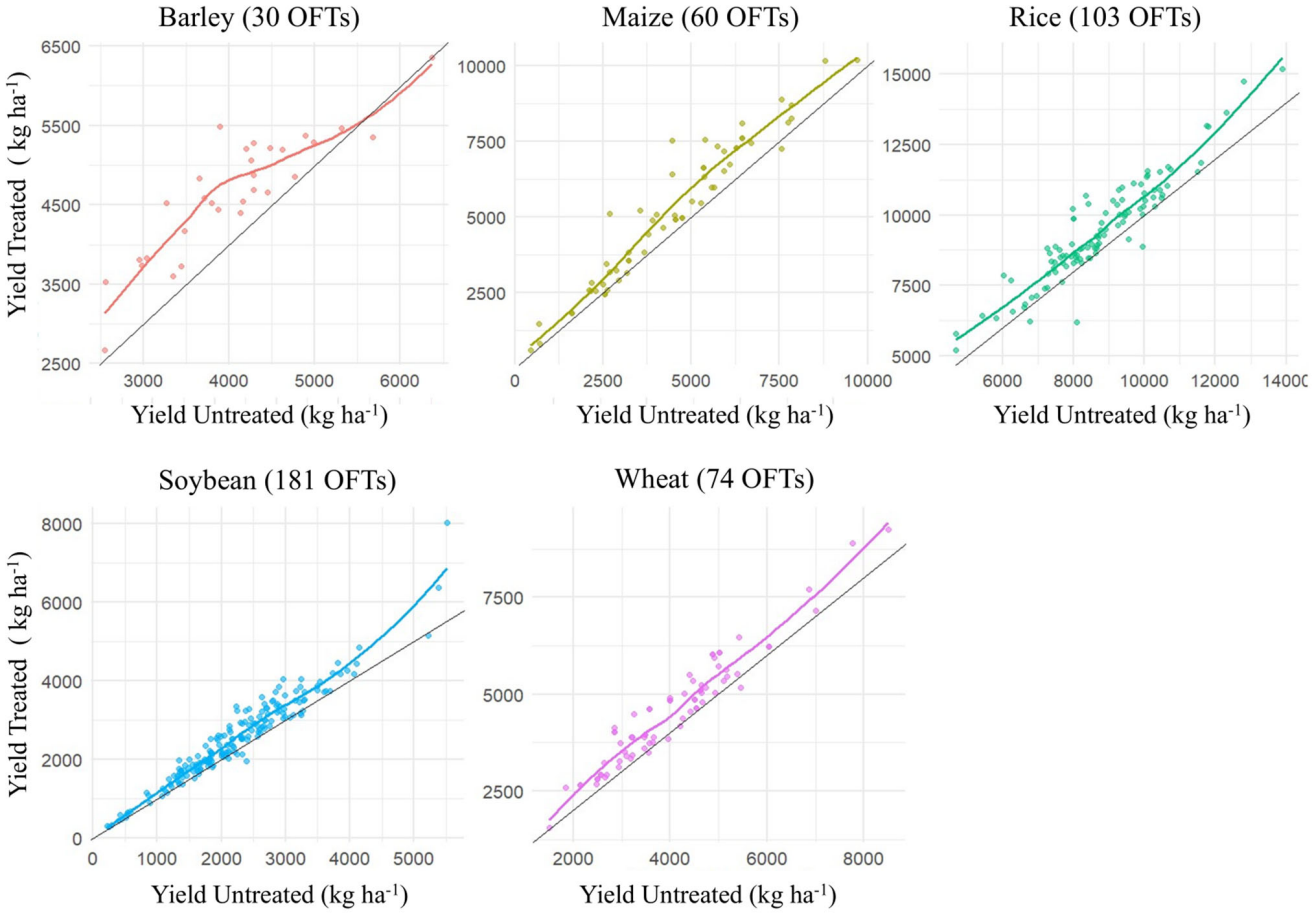
Yield responses of the five crops through the yields obtained in untreated OFTs. The colored curves represent the Loess regression. The black lines represent a simple linear regression relating the yield response to the yield in untreated OFTs. The horizontal line represents the OFT, where the yield response to the HB was zero.

**Figure 2:**
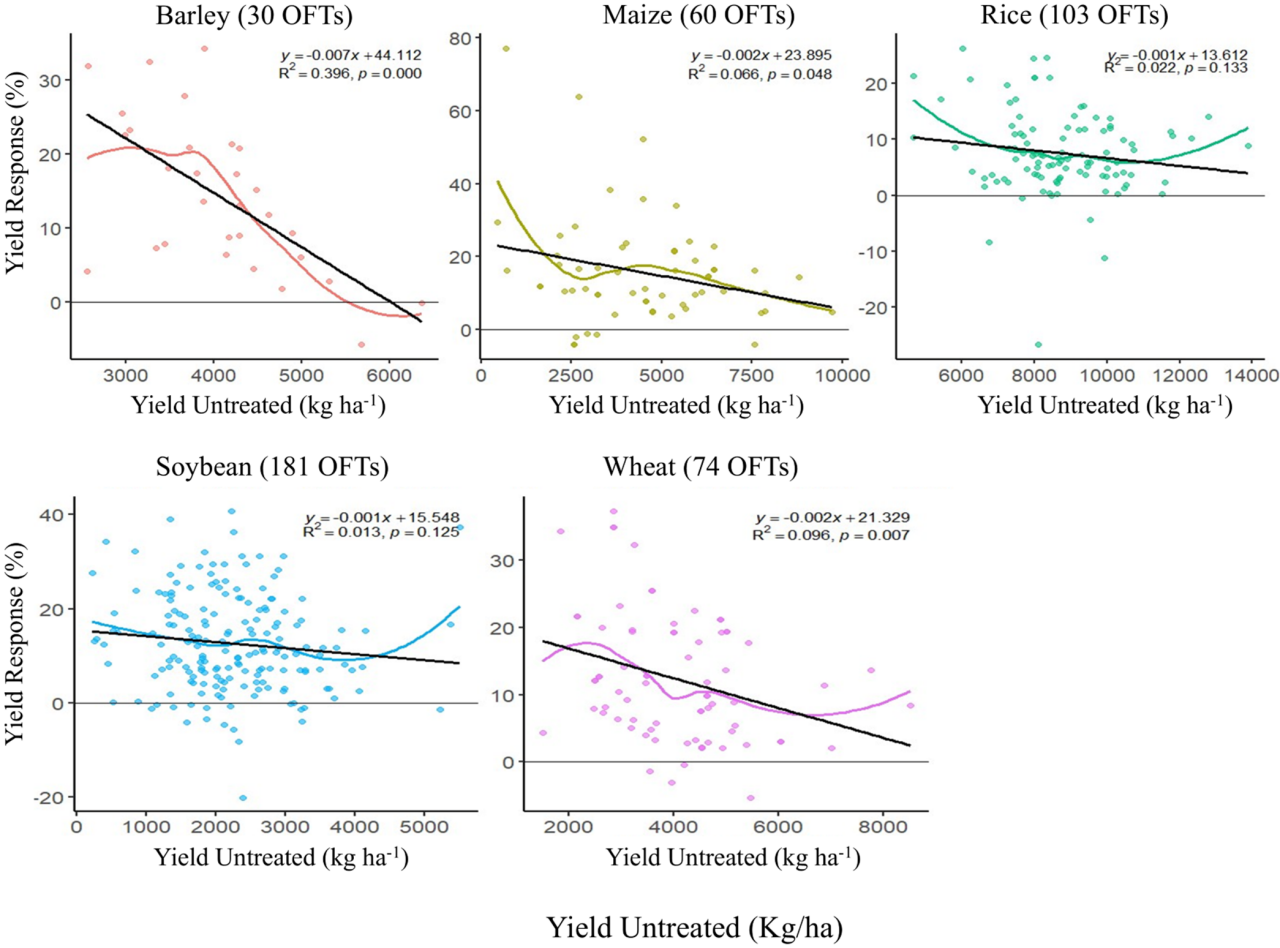
Scatter plots of yields for treated (y-axis) and untreated (x-axis) in all five crops. The colored curve represents the Loess regression used to evaluate the effect of humic biostimulant application on yield. The black lines represent the x = y function in the figure.

As expected, the ample diversity of environments, cultivars used, and farmers’ crop management practices for different crops and years resulted in a wide range of OFT untreated and treated mean yields. The overall mean yield of untreated OFT varied from 2.2 t ha^−1^ (ranging from 0.2 t ha^−1^ to 5.5 t ha^−1^) for soybeans to 8.7 t ha^−1^ (ranging from 4.6 to 13.8 t ha^−1^) for rice. Despite this, their overall means by grain crop across their respective periods were close to the country-wide mean grain yields for each of the five crops for those same years (**Table 2**). This result represents a reliable baseline for calculating the true crop response to a single application of HB, as proposed by Laurent et al. (2019). Similarly, the overall mean yield of treated OFT varied from 2.5 t ha^−1^ (ranging from 0.2 t ha^−1^ to 8.0 t ha^−1^) for soybeans to 9.4 t ha^−1^ (ranging from 5.1 t ha^−1^ to 15.1 t ha^−1^) for rice. The application of HB increased the yield of all five crops; however, its effect varied according to the different yields obtained under untreated OFT conditions. In barley, HB had a greater effect on lower-yielding untreated OFTs, reaching approximately 4 t ha^−1^. In rice, a tendency towards a greater effect in low- and high-yielding OFTs has been observed. In maize, wheat, and soybeans, HB had a relatively constant effect; however, a greater yield increase was observed among untreated OFTs, where the yield was ~5 t ha^−1^ for maize and wheat and ~3 t ha^−1^ for soybeans (**Figure 1**). These results were also evident according to the yield responses of the five crops (**Figure 2**). The overall mean yield response to the HB unique application, calculated using the mean yield response at each OFT for each crop, was significant according to the CI values and ranged from 7.6% in rice to 15.7% in maize (**Table 3**). According to the linear models, there was no significant effect of crops and year on yield response, except in 2023 (**Supplementary Table 4**). Furthermore, a trend toward a greater yield response in less productive OFT was observed according to simple linear regression. However, this trend was not significant for rice and soybeans (**Figure 2**). The yield of soybeans increased by 13.6% (**Table 2**) with a single application of HB applied in R3 at 4 L ha^−1^. As reported by Izquierdo et al. (2023), in our case, the yield increase by HB was due to increased pod retention (data not shown). The observed overall mean yield of the untreated strips (control) of 2.1 kg ha^−11^ in the 9 years reached 95.7% of the estimated national average yield of 2.2 t ha^−1^ (DIEA, 2023), thus demonstrating that HB is a practical amendment for improving soybean production for mainstream Uruguayan farmers. The OFT method can also discriminate the yield response under adverse seasonal conditions, such as droughts. Data from the Instituto Uruguayo de Meteorología (Inumet) showed that the 2020, 2021, and 2023 summer seasons had low rainfall and drought conditions Inumet (2023) (**Supplementary Table 3**). We found that in soybeans, the yield response was greater when the untreated control had very low yields during dry years (**Supplementary Figure 3**). However, in rainy years (2014 and 2019), the response was not related to the yield of untreated soybean crops. In rice, the overall mean yield response (7,56%) was significant (**Table 3**), with smaller decreases in those sites with the most appropriate conditions for cultivation (**Figure 2**). On 66% of the sites with good productivity, the untreated field yield was above 8 t ha^−1^, which meant a gain of over 650 kg ha^−1^ that will help to shorten the yield gap. In our case, one foliar application of HB at the wheat flag leaf growth stage resulted in an overall mean yield increase of 12,7% over 14 years, with a nonsignificant tendency to reduce response in locations with higher productivity (**Figure 2**). In barley, we found that a unique application of HB at the flag leaf growth stage resulted in an overall mean yield increase of over 14% in six years of OFTs (**Table 3**) and a significant tendency to reduce response at locations with higher productivity (**Figure 2**).

**Table 2:** Country and On-farm trials (OFT) untreated mean yields for soybean, rice, maize, wheat, and barley.

Crop	Period	Country mean yields	OFT untreated mean yield	Range untreated Yield	OFT treated mean yield	Range treated yield
		t ha^−1^				
Soybean	2014–2023	2.065	2.216	0.229–5.516	2.518	0.289–8.018
Rice	2015–2023	8.641	8.716	4.676–13.894	9.407	5.190–15.174
Maize	2019–2021	4.507	4.470	0.443–9.708	5.181	5.950–10.183
Wheat	2009–2023	3.595	4.050	1.496–8.509	4.557	1.563–9.256
Barley	2017–2023	3.820	4.058	2.557–6.369	4.642	2.664–6.359

*DIEA/MGA (2023) database. **Confidence interval (CI) is 95% for OFT untreated mean yield.

**Table 3:** Yield response and effectiveness of the HB application in grain crops at the main producing zones of Uruguay.

Crop	OFTs	Yield response % over the untreated control		Effectiveness	
		Mean	CI	>0%	>10%
Soybean	180	14.22	12.50–15.93	90.6	55.24
Rice	103	7.56	6.11–9.03	93.1	31.01
Maize	60	15.73	11.96–19.45	90.2	60.65
Wheat	74	12.71	10.48–14.99	94.6	50.1
Barley	47	14.29	10.67–17.97	93.3	53.3

CI, confidence interval at 95%.

The efficacy of humic products on maize can vary with environmental conditions, which could explain the inconsistent crop yield responses (Olk et al., 2021). We found that for the five crops, the linear regression model between the yield of the untreated control and the yield response (%) showed a negative trend; however, it was only significant (p <0.05) for barley, maize, and wheat (**Figure 2**). This result could indicate that the biostimulant has a greater effect on less productive conditions (varieties or soils) and less effect on the most productive conditions. In 448 OFTs installed between 2014 and 2024, 35 cases resulted in a negative response of grain yield to the HB.

### 3.2 Economic returns on grain crops

Considering a normal scenario, the net economic return varied from US $85.1 to $122.0 ha-1 for wheat and maize, respectively (**Table 4**). While the probability of exceeding the break-even cost ranged from 83.4% for soybean to 89.2% for wheat (**Table 5**). Interestingly, under an unfavorable scenario, the probability of exceeding the break-even cost was 79% and 81% for soybean and wheat, respectively. Therefore, this study demonstrates for the first time that a single application of HB at a critical time in the development cycle of these five crops can support the transfer of this simple technology to achieve higher yields and economic benefits for farmers, even when crop selling prices fall and the product price rises.

**Table 4:** Net economic return to the application of HB in grain crops.

Crop	OFT untreated mean yields (t ha^−1^)	Yield mean response to HB (%)	Net economic return to the HB application US$ ha^−1^
Soybean	2.216	14.22	92.0
Rice	8.716	7.56	118.6
Maize	4.470	15.73	122.0
Wheat	4.050	12.71	85.1
Barley	4.058	14.29	92.6

**Table 5:** Break-even yield and chance to exceed cost as a result of the application of an HB on grain crops under three market price scenarios. .

Crop	Scenarios	Price $US.t^−1^	Cost (product + application) $US.ha^−1^	Break-even yield (kg)	Chance to exceed cost (%)
Soybean	1	304.9	25.1	82.32	79.01
	2	384.2	24	62.47	83.43
	3	463.6	24	51.76	84.53
Rice	1	189.1	32.4	171.42	85.44
	2	216.6	31	143.12	86.41
	3	244.1	31	126.99	87.38
Maize	1	179.4	32.4	180.6	85
	2	215.2	31	144.05	88.34
	3	251.1	31	123.45	90
Wheat	1	169.8	25.1	147.82	81.09
	2	215.2	24	111.52	89.19
	3	260.5	24	92.13	93.25
Barley	1	166.3	25.1	150.93	83.33
	2	199.8	24	120.12	86.67
	3	233.4	24	102.82	90

Scenarios and Asumptions: (1) Unfavorable = Mean price − 1 SD; Cost (+4.5% inflation); (2) Normal = Mean price; costs; (3) Favorable = Mean price + 1 SD; costs.

## 4 Discussion

### 4.1 Yield increase by the HB application

The positive response to HB application in soybean in most OFTs and over an extensive period of years is very promising for this crop. Soybeans have established themselves as the main agricultural crop in Uruguay, both in terms of production and planted area. This legume, an oil and feed dual-purpose crop, represents 90% of the country’s area planted with summer crops and is the main export item of agriculture, contributing in 2022, with US$1,992 million of the US$13,556 million earned by agricultural exports (DIEA, 2023).

According to models of positive climatic trends in growing season precipitation, the country is among those with the largest potential to improve maize yield in South America (Markley, 2015). Our results showed an effectiveness of 90% and a response to HB of 15% (**Table 2**). These results agree with those obtained by Olk et al. (2022), who found responses of up to 4% with an effectiveness of 77% after the field foliar application of an HB in V6 at 35 production fields in Iowa, USA. The mitigation of water stress by humic acid has been reported (Canellas and Olivares, 2014; Chen et al., 2022). In our study, although not conclusive, the results are promising in terms of the ability of HB to provide greater stability in response to water deficit conditions. Uruguay’s mean wheat production set a record for grain yield despite the drought that prevailed in 2023. This was the result of genetic improvement in long-cycle and intermediate cultivars and exceptionally good climatic conditions (radiation and temperature in the period (flowering −20 + 10 days), adequate rainfall in the emergency-flowering period, pest control, fertilization, and early planting dates). The 2023–2024 wheat harvest reached a grain yield of 5 t ha^−1^, which is a historical maximum for the crop since 1995, when record-keeping began, over an area of 267,000 ha (DIEA/MGAP, 2024).

Yield gap assessments are key to developing management practices that sustainably increase rice production. Although rice is an important food crop worldwide, yield gaps in Latin American rice cropping systems remain under-investigated (Tseng et al., 2021). Field-level factors, such as cultivars, early seeding, N rate, and stand establishment, are influential in reducing yield gaps. The results of a four-year project sponsored by the Latin American Fund for Irrigated Rice (FLAR) and the National Agricultural Research Institute (INIA) of Uruguay show the need for integrated management (cattle and crops long rotation) to reduce the yield gap and achieve a yield of 10 t ha^−1^ (Gallego, 2023). Although these results represent an important advance in reducing the country yield gap of 2.4 t ha^−1^–3.2 t ha^−1^ (GYGA, 2024; www.yieldgap.org), there remains a need to further increase yields over a prolonged period.

High-quality barley cultivars are required in Uruguay. However, good grain malting quality is often linked to poor agronomic adaptation to abiotic stress, imposing a severe constraint on the development of the malting barley industry (Ceretta and van Eeuwijk, 2008). The crop growth stage that is most sensitive to water stress (either due to excess or deficit) is the stem elongation stage, followed by anthesis and grain filling. Water deficit around anthesis can lead to a loss in yield through the reduction in the number of ears and spikelets, which affects the fertility and survival of the spikelets. In contrast, a water deficit period during grain filling reduces the grain weight (Lamarca et al., 2010). Barley is particularly sensitive to excess water (waterlogging), which affects its yield. Water stress for 8 days during tillering on different cultivars resulted in a 30% reduction in yield per plant (Hoffman et al., 2008).

Adverse results with HB have been associated with incorrect application, timing, and dose in field trials and commercial farming (de Santiago et al., 2010; Fleming et al., 2019). However, these may be due to natural variation, equipment problems, greater tiller induction in rice cultivars susceptible to the fungus *Pyricularia Oryza* without appropriate pesticide control, flooding, or extreme heat (Izquierdo et al., 2023). In our case, all responses (including negative ones) in the five crop data files were considered in the response analysis because they often show an important source of yield variability (Laurent et al., 2019).

### 4.2 Economic benefits

A major gap in the widespread use of HBs is related to their reliability over time and space in benefiting grain crop yield. Humic products do not appear to promote crop growth in all situations, given the variable results reported thus far (Olk et al., 2021). Thus, there is a need to determine whether there is a predictable pattern in when and where humic products improve crop growth and provide economically viable returns. The integration of biostimulants into agricultural practices largely depends on their economic importance compared to conventional practices, as their application may be economically beneficial for one farm and useless for another. To establish the economic efficiency of biologically active stimulants, economic-mathematical models based on linear optimization can be applied to assess not only the increase in profitability and profit of certain crops but also whether the profit of the farm is increasing (Sarov and Boevsky, 2021). Therefore, the benefits must be demonstrated in the form of research results and educational and transfer programs that focus on real data obtained from field experiments or on-farm trials. Based on these results, the HB was registered with the Ministries of Agriculture of Uruguay and Brazil. Its use in Uruguay reaches 70% of rice producers and an estimated 75,000 ha of other grain crops. The economic effect of biostimulant application must be computed based on the value of the yield increase resulting from the use of biostimulants and the costs of their application (Kocira et al., 2020). In our case, the empirical cumulative distribution functions for the five crops used to estimate the break-even yield response and the probability of expected profit were useful, as proposed by Laurent et al. (2020b), and this finding was also confirmed by the sensitivity test with three market price scenarios.

### 4.3 Humic biostimulant use and perspectives

Humic substances (HS) promote yield gains through direct biostimulation, including improved nutrient availability and uptake, stimulation of plant growth pathways, and mimicking plant hormones such as auxins, which stimulate root growth and boost metabolic processes such as photosynthesis and respiration (Maffia et al., 2025). The mechanistic explanations of HS direct plant biostimulation have been shown to increase plant height and dry or fresh weight and enhance nutrient uptake (El-Tahlawy and Osama, 2022). HS affects enzyme activity, protein metabolism, photosynthesis, respiration, and water and nutrient uptake. The underlying mechanisms involve hormone fluxes, cell membrane permeability, electron chain transport components, free radical activity within the humic structure, and reactive oxygen species in plants (Das et al., 2025). The complex hormonal crosstalk networks in plants are an example of a precisely calibrated regulatory system that strikes a balance between growth and abiotic stress adaptation. Auxins, particularly indole-3-acetic acid (IAA), play a key role in plant growth and influence cell elongation, division, and differentiation. When plants face abiotic stress, auxin production, movement, and signaling change, which affect root structure and stress responses (Jing et al., 2023). Cytokinins play complex and multifaceted roles in enhancing plant tolerance to drought and salinity, which are two major abiotic stresses affecting global crop productivity (Mughal et al., 2024). The specific modes of action of HB are currently under investigation. Humic substances are highly complex and biologically active and can mitigate stress damage effects by mediating signaling pathways that involve different plant hormones (Canellas and Olivares, 2014). Their role in plant nutrition goes beyond the increase in plant root morphology, and these substances can form a complex with cations present in the soil, improving the uptake of nutrients such as P, Zn, and Fe by plants (Olaetxea et al., 2018; Nardi et al., 2021). Although there is a consensus on the potential benefits of the interaction between HB and crops, there is still limited scientific evidence for grain crops under field conditions underpinning this interaction. Nevertheless, most studies on the physiological responses to humic applications have been conducted under controlled conditions (greenhouses, pots, *in vitro*, and growth chambers) (Calvo et al., 2014). Positive yield responses to HB under field conditions have been obtained for soybean (Prado et al., 2016; Izquierdo and García-Pintos, 2021; Izquierdo et al., 2023), rice (Talha et al., 2020; Izquierdo et al., 2021; 2024), maize (Efthimiadou et al., 2020; Olk et al., 2022), wheat (Pacuta et al., 2021), malting barley (Abdel-Ati et al., 2023), and rapeseed (Passandideh et al., 2023). Humic plant biostimulants differ from other agricultural inputs, such as fertilizers and plant protection products, because they utilize different mechanisms and work regardless of the presence of nutrients in the products. They also do not take any direct action against pests, thus complementing the use of fertilizers and plant protection products. A meta-analysis of humic substances under controlled environment and field studies reported a 20% increase in dry weight of shoots and roots (Rose et al., 2014), and an average yield gain of 17.9% was found for biostimulants, showing the strongest crop yield response in soils of low quality (Li J. et al., 2022). These results are in agreement with the range of response sizes to HB in cereal crops under field conditions. For biostimulants to become standard practice, these products require consistency and reproducibility in their effects on crop production. Emerging technologies that address these critical problems include the development of novel plant biostimulants and successful methods for their application (Hamid et al., 2021), as well as effective analysis and summarization of on-farm study outcomes and communication of results to farmers and agronomists. In our case, a detailed report was prepared for the farmers at each OFT, including graphics and statistical summaries of yield differences showing the farm, year, and zone results.

## 5 Conclusions

Over the past 30 years, various technical advancements have been proposed to boost sustainable agricultural production and minimize the use of synthetic products, such as pesticides and fertilizers. On-farm trials are a useful method for assessing and expanding sustainable practices among farmers, considering that this bio-input is produced domestically through waste material recycling. The application of a humic biostimulant at key growth stages is suitable for enhancing grain crop yields, ranging from 7.6% in rice to 15.7% in maize. Our results show that economic gains from various crops varied positively, ranging from $68 per hectare in wheat to $164 per hectare for rice, with the probability of surpassing the break-even point spanning 79.7% for wheat to 87.5% for rice. This demonstrates, for the first time, that a single foliar application of HB at a critical stage in the developmental life cycle of these five grain crops can facilitate the adoption of this straightforward, eco-friendly, and low-cost technology to boost yields and economic returns for Uruguayan farmers and potentially others abroad. Investigations into the physiological mechanisms of grain crops and biostimulant gene signaling, coupled with field studies of parameters such as leaf chlorophyll evolution and nitrogen dynamics during the reproductive stages, hold the potential for gaining a more in-depth understanding of the underlying mechanisms governing the response to humic biostimulants.

## Data Availability

The original contributions presented in the study are included in the article/**Supplementary Material**. Further inquiries can be directed to the corresponding author.
