# Supplementary material for: Humic field biostimulation as a sustainable agricultural practice to increase yield of main grains: evidence from on-farm trials
Source: Front Plant Sci. 2025 Dec 9;16:1709876. doi: 10.3389/fpls.2025.1709876 (PMC12723518; doi:10.3389/fpls.2025.1709876)
Supplement: Supplementary file 2 [file Supplementaryfile1.docx]

SUPPLEMENTARY MATERIAL

## Supplementary Figures


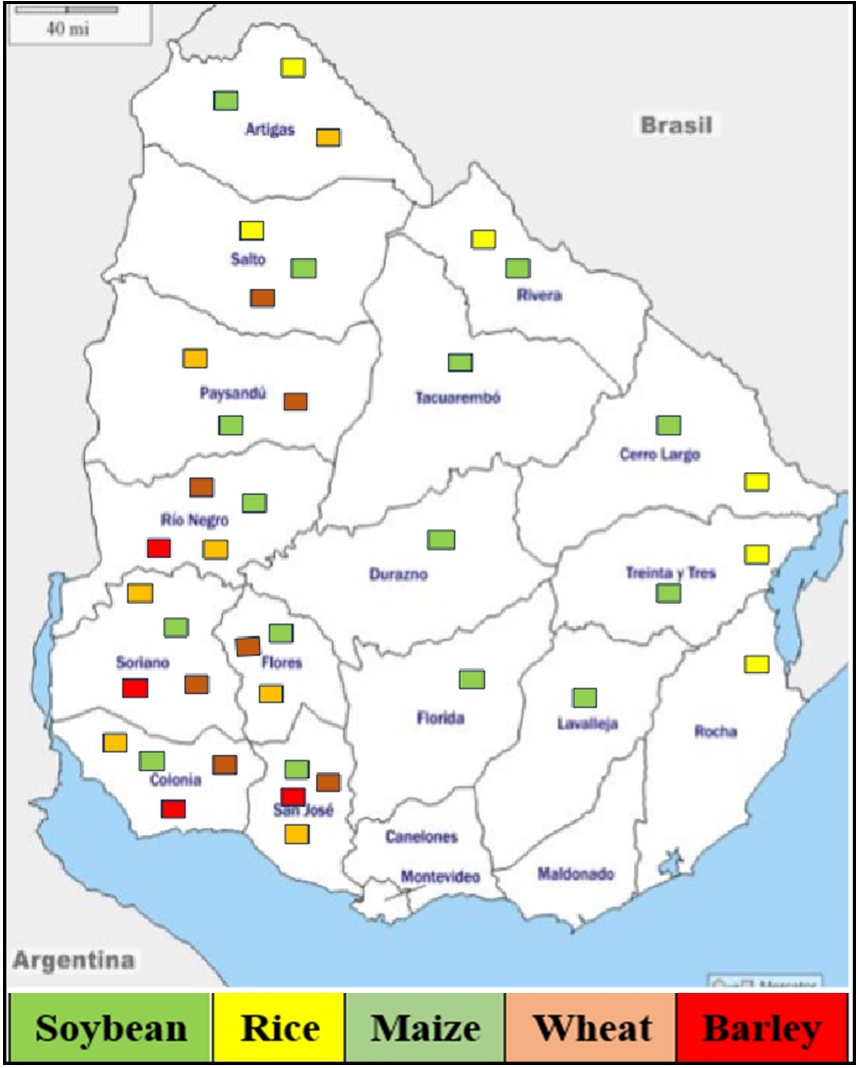


**Supplementary Figure 1**. Departments of Uruguay where on-farm trials of soybeans, rice, maize, wheat, and barley were conducted from 2014 to 2023.


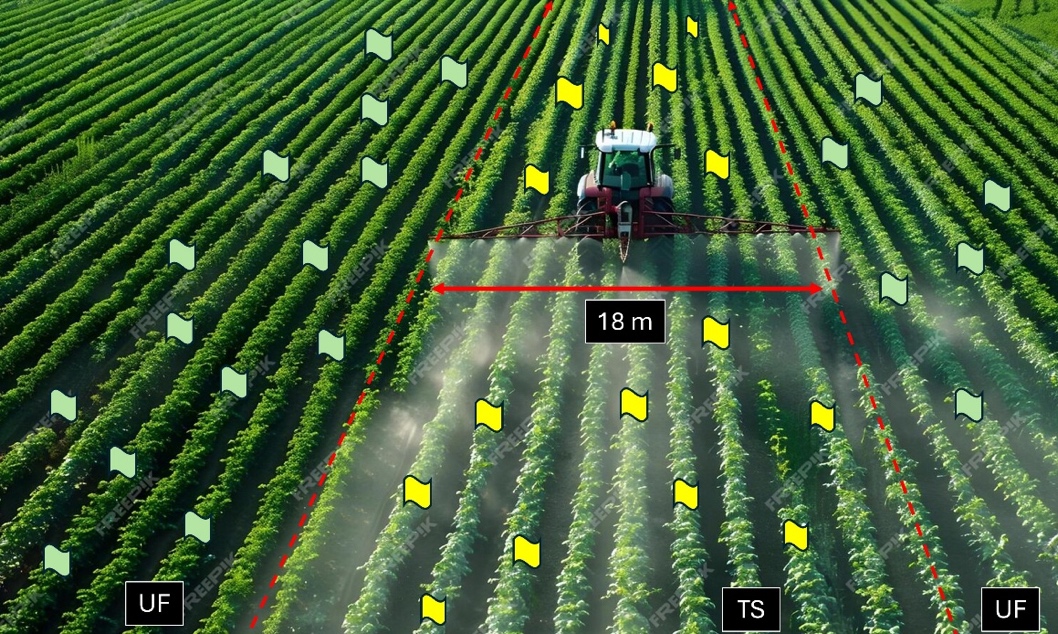


**Supplementary Figure 2.** HB spraying on the treated strip (TS) installed across an OFT untreated field (UF) and random row samples to be collected at maturity. The image was generated with Artificial Intelligence and extracted from <https://www.freepik.com/>


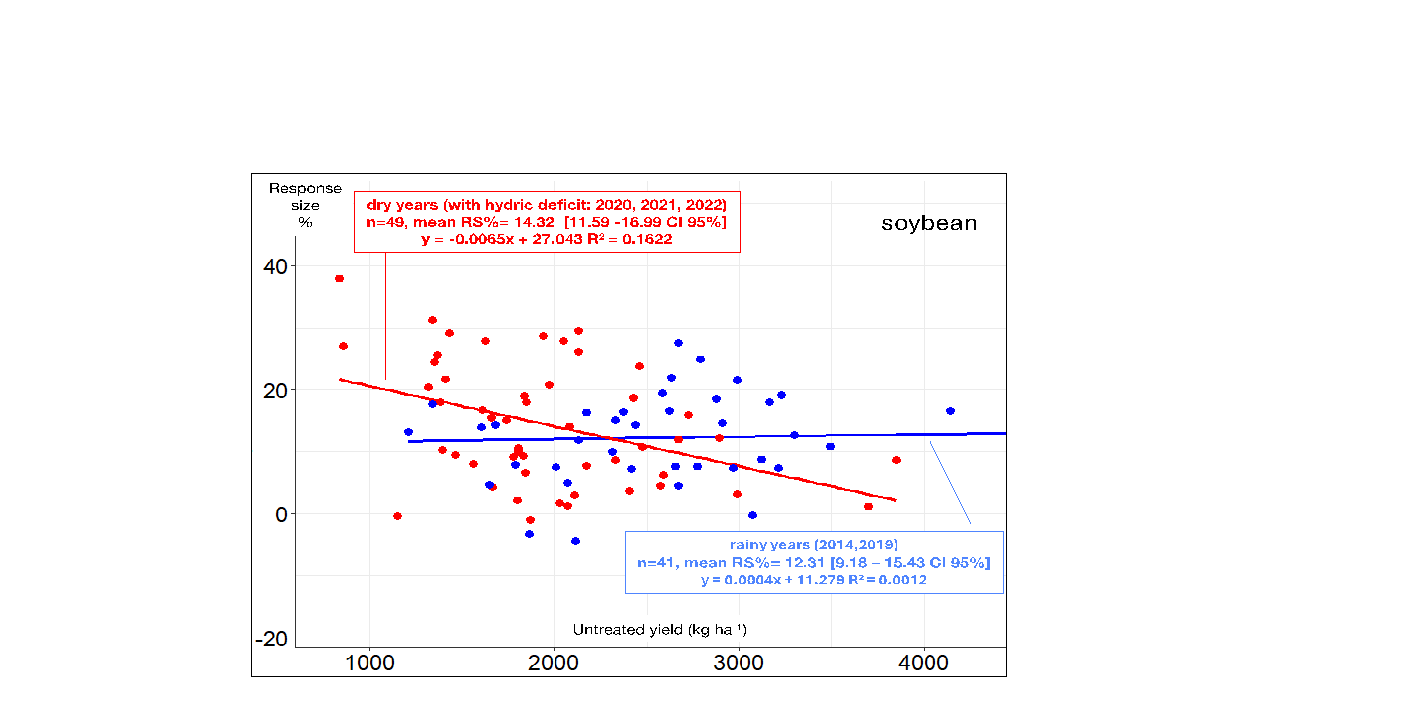


**Supplementary Figure 3.** Soybean yield response over the untreated control when HB was applied at OFTs conducted in Uruguay in drought-season years (2020, 2022, and 2023 [red dots; n=49] and rainy-season years (2014 and 2019 [blue dots; n=41]).

## Supplementary Tables

## Supplementary Table 1. Key characteristics of soils, cultivars used, and predominant agronomic management of on-farm trials conducted in Uruguay.

| **Crop** | **Key characteristics of OFT farm soils** | **Main cultivars used** | **Predominant agronomic management by farmers.**  . |
| --- | --- | --- | --- |
| **Soybean** | Brunosols and Lithosols, soils with clay loam to silty clay loam textures. Dark, deep, and fertile. They have a loamy texture, are well-drained, and rich in organic matter, with a pH between 6.0 and 7.5, not compacted and with good drainage. | Cultivars of maturity groups V to VII. | Direct sowing, plant population 250,000/ha, row spacing 0.38 m. Fertilization: 70 P_2_O_5_ kg ha^-1^, 10 S kg ha^-1^, herbicides, pest and disease control. |
| **Rice** | Planosols, Gleysols, Lithosols, and soils developed on basalt, flat or with gentle slopes. Clay-loam or silt-loam texture at A horizon, high water-holding capacity, and high clay and organic matter content. Moderately acidic or neutral pH. | INIA Merín  Gurí INTA  INIA Olimar  INIA Tacuarí INOV | Recommendations from the National Institute of Agricultural Research (INIA) and Rice Growers Association (ACA) on integrated crop management including crop rotation with pastures, soil preparation, water management, balanced fertilization, weed, pest and disease control, and proper harvesting and post-harvest management. |
| **Maíze** | Brunosols with sandy clay loam to sandy loam texture and black and red Lithosols, with silty clay loam textures | Yellow hybrids (dent) for industrial processing and animal feed. | Recommendations from the National Institute of Agricultural Research (INIA) on plant density, fertilization, herbicides, weeds, pest and disease control. |
| **Wheat** | Brunosols with sandy clay loam to sandy loam texture and black and red Lithosols, with silty clay loam textures | INIA Torcaza, INIA Gorrión INIA Tijereta, INIA Kipa, INIA Millán | Early sowing. Planting density depends on the variety and soil. Fertilization: 50 N kg ha^-1^and 20 S kg ha^-1^ at sowing, and 15-25 N P kg ha^-1^ at tillering.  Herbicides for weed control. |
| **Barley** | Well-drained loamy, clayey, or marly soils with a pH between 6 and 7.5. Brunosols with a sandy clay loam to sandy loam texture. Shallow black Lithosols. | INIA Arrayán, INIA Osiris Norteña Carumbé, Norteña | Balanced N, P, and K fertilization. 50 N kg ha^-1^ at sowing and 15 - 25 N P kg ha^-1^ at tillering. Planting density: 116 kg/ha. Herbicides for weed control. |

**Supplementary Table 2**. Average maximum temperature °C, Penman evapotranspiration (mm), and effective precipitation (mm) of February, March, and April, from 2014 to 2023. Data: INIA GRAS, La Estanzuela Experimental Station, Uruguay. Light blue year-months present a water deficit.

| Year | Month | Average  maximum air temp.  (ºC) | Potential evapotranspiration^1^  (mm) | Effective Precipitation^2^ (mm) |
| --- | --- | --- | --- | --- |
| 2014 | Feb | 26,05 | 105,8 | 222,6 |
|  | Mar | 24,27 | 102,2 | 114,8 |
|  | Apr | 21,88 | 68,5 | 68 |
| 2015 | Feb | 28,25 | 137,3 | 25,1 |
|  | Mar | 27,07 | 119 | 25,8 |
|  | Apr | 25,24 | 94,8 | 45,1 |
| 2016 | Feb | 30,11 | 178,1 | 124,3 |
|  | Mar | 24,47 | 141,6 | 84,2 |
|  | Apr | 20,04 | 94,3 | 208,5 |
| 2017 | Feb | 28,59 | 118,3 | 68,2 |
|  | Mar | 26,18 | 103,2 | 112,7 |
|  | Apr | 22,83 | 64,8 | 42,7 |
| 2018 | Feb | 28,81 | 147,4 | 60,6 |
|  | Mar | 27,26 | 123 | 103,1 |
|  | Apr | 25,31 | 59,7 | 103,8 |
| 2019 | Feb | 27,57 | 122,4 | 102,7 |
|  | Mar | 24,73 | 89 | 82,8 |
|  | Apr | 23,89 | 66,7 | 20,8 |
| 2020 | Feb | 28,69 | 158,4 | 61,5 |
|  | Mar | 25,93 | 121,3 | 57,6 |
|  | Apr | 22,31 | 69,8 | 110,4 |
| 2021 | Feb | 26,72 | 112,6 | 103,8 |
|  | Mar | 25,25 | 91,9 | 83,8 |
|  | Apr | 24,14 | 64,5 | 76,9 |
| 2022 | Feb | 26,72 | 129,1 | 93,2 |
|  | Mar | 24,44 | 110 | 83,4 |
|  | Apr | 21,7 | 71,3 | 77,3 |
| 2023 | Feb | 30,77 | 170,7 | 5,4 |
|  | Mar | 30,93 | 157,5 | 41,3 |
|  | Apr | 23,52 | 40,1 | 23,8 |
| 1 Potential evapotranspiration according to the Penman-Monteith method.  2 Effective Precipitation (mm)= daily precipitation, excluding surface runoff. | | | | |

**Supplementary Table 3.** Uruguayan grain market price of grain crops from 2014 to 2023

| Crop | 2014 | 2015 | 2016 | 2017 | 2018 | 2019 | 2020 | 2021 | 2022 | 2023 | Mean SD | |
| --- | --- | --- | --- | --- | --- | --- | --- | --- | --- | --- | --- | --- |
|  | (USD. t^-1^) | | | | | | | | | |  |  |
| Soybean | 365 | 335,36 | 366,88 | 342,36 | 351,28 | 310,15 | 338,91 | 488,05 | 564,31 | 380 | 384,23 | 79,3479 |
| Rice | - | 217,97 | 205,01 | 192,88 | 187,17 | 187,97 | 214,71 | 238,46 | 234,97 | 270 | 216,57 | 27,5115 |
| Maize | - | 196,07 | 207,1 | 189,4 | 226,29 | 177,79 | 206,4 | 256,48 | 287,56 | 190 | 215,23 | 35,8332 |
| Wheat | - | 195,93 | 153,73 | 183,96 | 215,67 | 217,89 | 207,99 | 250,92 | 313,35 | 197 | 215,16 | 45,3125 |
| Barley | - | 227,71 | 198,01 | 172,1 | 194,04 | 164,97 | 170,01 | 214,76 | 269,97 | 187 | 199,84 | 33,5310 |

Source: MGAP-DIEA, 2023 based on information collected from local grain traders. The average interbank dollar value for each year was considered.

**Supplementary Table 4**. Linear models to assess whether the average yield response to the application of the biostimulant varies significantly depending on the crop and the year.

|  |  | **Source of variation** | **Df** | **Sum Sq** | **Mean Sq** | **F value** | **Pr(>F)** |
| --- | --- | --- | --- | --- | --- | --- | --- |
| Crops | Barley | Year | 5 | 0,04986 | 0,009972 | 0,925 | 0,482 |
|  | Maize | Year | 7 | 0,2354 | 0,03362 | 1,627 | 0,148 |
|  | Rice | Year | 8 | 0,0266 | 0,003328 | 0,567 | 0,802 |
|  | Soybean | Year | 9 | 0,0295 | 0,003278 | 0,297 | 0,975 |
|  | Wheat | Year | 7 | 0,0711 | 0,01015 | 1,143 | 0,347 |
| Years | 2015 | Crop | 3 | 0,0268 | 0,008931 | 0,607 | 0,614 |
|  | 2016 | Crop | 1 | 0,00194 | 0,001935 | 0,237 | 0,631 |
|  | 2017 | Crop | 2 | 0,00947 | 0,004737 | 0,616 | 0,547 |
|  | 2018 | Crop | 2 | 0,0356 | 0,01779 | 1,759 | 0,186 |
|  | 2019 | Crop | 2 | 0,0233 | 0,01167 | 0,808 | 0,452 |
|  | 2020 | Crop | 3 | 0,02921 | 0,009736 | 1,695 | 0,186 |
|  | 2021 | Crop | 4 | 0,0561 | 0,014021 | 2,217 | 0,0805 |
|  | 2022 | Crop | 4 | 0,0726 | 0,018158 | 1,857 | 0,138 |
|  | 2023 | Crop | 4 | 0,3188 | 0,07969 | 4,635 | 0,00371 |
